# P-1066. Quorum Sensing Coordinates Phage Defense Mechanisms in *Pseudomonas aeruginosa*

**DOI:** 10.1093/ofid/ofae631.1254

**Published:** 2025-01-29

**Authors:** Dandan Li, Na Li, Yu Chen, Yuxuan Yang, Jue Pan, Jiabing Lin, Xiaodong Gao, Rong Bao, Chunmei Zhou, Suzhen Wang, Bijie Hu, Demeng Tan

**Affiliations:** Zhongshan Hospital, Fudan University, Shanghai, Shanghai, China (People's Republic); Zhongshan Hospital, Fudan University, Shanghai, Shanghai, China (People's Republic); Department of Infectious Diseases, Zhongshan Hospital, Fudan University, Shanghai, Shanghai, China; Zhongshan Hospital, Fudan University, Shanghai, Shanghai, China (People's Republic); Zhongshan Hospital, Fudan University, Shanghai, Shanghai, China (People's Republic); Zhongshan Hospital, Fudan University, Shanghai, Shanghai, China (People's Republic); Zhongshan Hospital of Fudan University, Shanghai, Shanghai, China; Zhongshan Hospital, Fudan University, Shanghai, Shanghai, China (People's Republic); Fudan university Zhongshan hospital, Shanghai, Shanghai, China; Zhongshan Hospital, Fudan University, Shanghai, Shanghai, China (People's Republic); Department of Infectious Diseases, Zhongshan Hospital, Fudan University, Shanghai, Shanghai, China; Shanghai Public Health Clinical Center, Shanghai, Shanghai, China

## Abstract

**Background:**

Quorum sensing (QS) plays a crucial role in regulating key traits, including the upregulation of phage receptors, leading to heightened phage susceptibility in *Pseudomonas aeruginosa*. Consequently, elevated cell densities usually escalate the likelihood of phage invasions. This observation has sparked speculation that bacteria might have evolved multiple antiphage defense mechanisms to offset the cost of increased phage receptor expression during the prolonged coevolution or cohabitation with phages.

The impact of cell-free supernatant on phage-host interactions in liquid medium was investigated.

**Figure:**
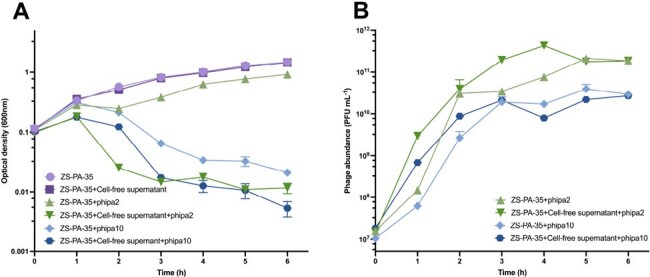

(A) Growth curves of ZS-PA-35 and (B) corresponding phage concentrations measured in plaque forming units (PFU) per milliliter, in the presence or absence of phage phipa2 or phipa10 at Multiplicity of Infection (MOI) of 2. Measurements were taken at 1-h intervals over a 6-hincubation period in conditioned medium. The conditioned medium was derived from high-cell density cultures of strain ZS-PA-35 and supplemented with 25% 4 × LB medium. Error bars depict standard deviations (n=3).

**Methods:**

The role of QS in defense strategies employed by *P. aeruginosa* to fight against phages phipa2 and phipa10’s predation was determined by comparing phage inhibition, adsorption, and resistance assays between ZS-PA-35 and its QS mutants (Δ*lasR*, Δ*rhlR*, Δ*lasI*, Δ*rhlI*, Δ*lasR* Δ*rhlR*, and Δ*lasI* Δ*rhlI*) at low- and high-cell-densities.

QS regulates phage-host interactions in liquid medium.

**Figure:**
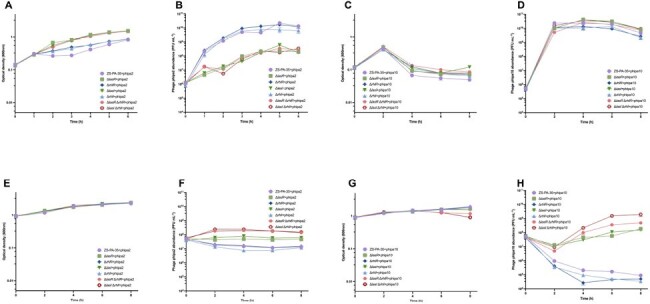

In LCD cultures, optical densities (OD600) and corresponding phage concentrations of P. aeruginosa wild-type (ZS-PA-35) and QS mutants (ΔlasR, ΔrhlR, ΔlasI, ΔrhlI, ΔlasR ΔrhlR, and ΔlasI ΔrhlI) were determined with phage phipa2 or phipa10 at MOIs of 0.01. In HCD cultures, similar measurements were taken at 2-h intervals over an 8-h period. Error bars represent standard deviations (n=3).

**Results:**

The Las system increases phage adsorption rates by upregulating the expression of Type IV pilus and O-antigen receptors, which subsequently leads to accelerated bacterial lysis. Moreover, the Las system, apart from mediating receptor mutations, orchestrates an additional intracellular defense mechanism to suppress the production of both phage phipa2 and phipa10, resulting in a reduction of over 2 logs in high cell density cultures of strain ZS-PA-35 and phipa10. These flexible regulatory systems optimize the function of various defense strategies, particularly when bacterial communities are most susceptible to phage invasion.

QS regulates phage adsorption.

**Figure:**
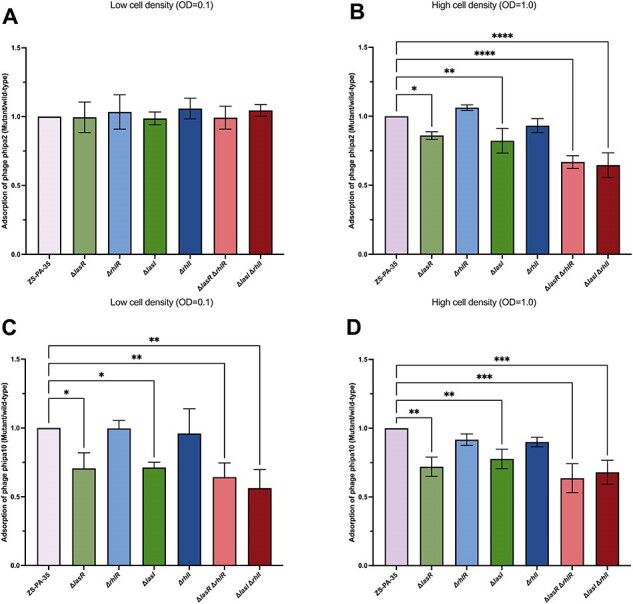

Adsorption assays were conducted to assess the interaction between phage phipa2 (A and B) or phipa10 (C and D) and its host strain ZS-PA-35, as well as QS mutants, under low cell density (LCD) conditions (A and C) and high cell density (HCD) conditions (B and D). The data represent averages from three independent samples, with error bars indicating standard deviations. Statistical significance was determined using ordinary one-way ANOVA with Dunnett’s test, with significance levels indicated as *P < 0.05, **P < 0.01, ***P < 0.001, and ****P < 0.0001. Significant results are highlighted accordingly.

**Conclusion:**

Bacteria employ QS to finely tune various defense strategies, both mutational and non-mutational defense mechanisms, highlighting the intricate nature of phage-host interactions and the challenges associated with the potential clinical use of phages as therapeutic agents.

**Figure:**
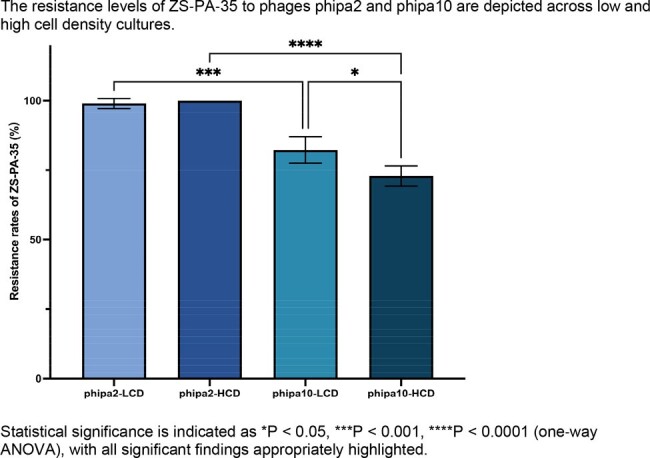

Statistical significance is indicated as *P < 0.05, ***P < 0.001, ****P < 0.0001 (one-way ANOVA), with all significant findings appropriately highlighted.

**Disclosures:**

**All Authors**: No reported disclosures

